# Fungal diversity in oil palm leaves showing symptoms of Fatal Yellowing disease

**DOI:** 10.1371/journal.pone.0191884

**Published:** 2018-01-25

**Authors:** Ohana Yonara de Assis Costa, Daiva Domenech Tupinambá, Jessica Carvalho Bergmann, Cristine Chaves Barreto, Betania Ferraz Quirino

**Affiliations:** 1 Embrapa-Agroenergy, Brasília, Distrito Federal, Brazil; 2 Embrapa-Sede, Brasília, Distrito Federal, Brazil; 3 Genomic Sciences and Biotechnology Program, Universidade Católica de Brasília, Brasília, Distrito Federal, Brazil; Oklahoma State University, UNITED STATES

## Abstract

Oil palm (*Elaeis guineensis* Jacq.) is an excellent source of vegetable oil for biodiesel production; however, there are still some limitations for its cultivation in Brazil such as Fatal Yellowing (FY) disease. FY has been studied for many years, but its causal agent has never been determined. In Colombia and nearby countries, it was reported that the causal agent of Fatal Yellowing (*Pudrición del Cogollo*) is the oomycete *Phytophthora palmivora*, however, several authors claim that Fatal Yellowing and *Pudrición del Cogollo* (PC) are different diseases. The major aims of this work were to test, using molecular biology tools, Brazilian oil palm trees for the co-occurrence of the oomycete *Phytophthora* and FY symptoms, and to characterize the fungal diversity in FY diseased and healthy leaves by next generation sequencing. Investigation with specific primers for the genus *Phytophthora* showed amplification in only one of the samples. Analysis of the fungal ITS region demonstrated that, at the genus level, different groups predominated in all symptomatic samples, while *Pyrenochaetopsis* and unclassified fungi predominated in all asymptomatic samples. Our results show that fungal communities were not the same between samples at the same stage of the disease or among all the symptomatic samples. This is the first study that describes the evolution of the microbial community in the course of plant disease and also the first work to use high throughput next generation sequencing to evaluate the fungal community associated with leaves of oil palm trees with and without symptoms of FY.

## Introduction

World economic growth has led to an increase in energy consumption. Fortunately, concern about environmental impacts is also increasing, prompting the development of cleaner and renewable energy sources. For this, investment in new sources of energy able to balance economic viability and sustainability is needed [1–3].

Brazil is a leader in the bioenergy sector, with an energy matrix largely based on clean, renewable sources. In 2016, approximately 44% of the Brazilian energy matrix was based on renewable energy sources, such as sugarcane ethanol and wind energy [4, 5]. The maintenance of this clean energy matrix has stimulated research on biofuels such as biodiesel produced from vegetable oils.

Due to its high oil yield per hectare, oil palm stands out as a potential source of oil for large-scale biodiesel production in Brazil. While coconut, soybean, sunflower and other crops have oil yields between 120 and 1,200 kg/ha, oil palm oil production ranges between 2,000 and 8,000 kg/ha [6], being considered the oleaginous crop with highest productivity.

Oil palm (*Elaeis guineensis* Jacq.) is a perennial plant native to West Africa. In Brazil, crop yield in 2014 was 11013.6 kg/ha and 370,000 t of oil were produced. Indonesia is the world top producer of palm oil [7]. Even though oil palm is an excellent source of vegetable oil for biodiesel production, there are still some limitations for its cultivation in Brazil such as Fatal Yellowing (FY) [6].

FY is a bud rot disease with variable symptoms that caused vast destruction on plantations in Central and South America. Thousands of hectares of oil palms were devastated in Brazil, Ecuador, Colombia, Costa Rica, Nicaragua, Panama and Suriname [8, 9]. In general, first symptoms consist of light yellowing of the basal leaflets of intermediate leaves (3, 4, 5 and 6) followed by necrosis of the borders, later extending to the remaining areas of the leaves [10]. Wet or dry rot spreads by contact from one leaflet to the next. The rot then spreads towards the spear leaf and growth point, frequently killing the oil palm trees [8, 9]. Nevertheless, symptoms and severity of the disease vary depending on the region. The disease appears to show different rates of development. For instance, it shows an acute form in Ecuador and southwestern Colombia, where it takes 1–2 months to kill the plants, whereas in Brazil and Suriname the death of the oil palm trees takes 1–3 years [9]. The spatial development also demonstrates variation. In some cases, FY starts with a linear spreading that later turns into an exponential dispersion. In other cases, such as in the temporal and spatial analysis performed by Bergamin Filho et al. [11] and Laranjeira et al. [12], progression of FY does not show a consistent development pattern.

It is important to note that several authors claim that FY and *Pudrición del Cogollo* (PC) are different diseases with different symptoms and evolution. Ayala [13] states that these diseases are different since a fetid rot meristem was not observed in oil palm trees in Pará state (Brazil). Swinburne [14] compared symptomatic cases among plants in Brazil, Colombia and Ecuador, and observed differences in disease evolution. For instance, a wet rot meristem and recovery of diseased plants do not occur in Brazilian plants. Zadoks [15] found that the symptoms of *Pudrición del Cogollo* in the Ecuadorian Amazon are different from the symptoms of FY in Brazil, although both can be lethal.

Even though FY has been studied for many years, its cause has never been determined, and no consistent correlations with specific insects, physiological problems, soil type or pathogens were found [10]. Moreover, there is no treatment available to control the disease in affected plants.

Martinez et al. [16], however, claimed that the causal agent of FY (*Pudrición del Cogollo*) in Colombia and nearby countries is the oomycete *Phytophthora palmivora*. In their studies [16, 17], *P*. *palmivora* was isolated from oil palm trees in the early stages of the disease using fruit traps. However, a molecular analysis to verify the presence of *P*. *palmivora* in the microbiota of diseased plants was not performed.

The aim of this work was to investigate in Brazilian oil palm trees with and without symptoms of Fatal Yellowing the presence of the oomycete *Phytophthora* using molecular biology tools, and to study the fungal diversity associated with the leaves of the same plants by next generation sequencing.

## Material and methods

### Sampling

Leaf samples were collected in State of Pará, Brazil, in an oil palm- cultivated area near the city of Mojú. No animals were involved in this study. The study was performed using samples from private land with permission from land owners. Samples were obtained from oil palm plants with Fatal Yellowing (FY Symptomatic Plants—SP), and from oil palm plants in a region with no occurrence of Fatal Yellowing (FY Asymptomatic Plants—AP). Plants without symptoms of FY were 4 km away from the symptomatic plants. Leaves were collected from symptomatic plants in the stages 2, 5 and 8 of the disease. The classification of disease into stages 1–10 was proposed by Souza et al. [18]; stage 1 is the start of the first visible symptoms of the disease and stage 10 is the most severe symptoms. In stage 2, young leaves are pale green and the shoot starts to wilt. In stage 5, necrosis starts in the tips of young and yellowish leaves. In stage 8, the oldest leaves are still dark green, but young leaves are yellow and pale green, and most of the leaves are already dry. The geographical coordinates of sampling points are shown in Table 1. Samples were collected from the second apical leaf of palm oil trees, from which 10 leaflets were removed near the apex, 10 from the middle section of the leaf and 10 from the base of the leaf. Leaflets were transported in a cooler containing dry ice. In the lab, samples were maintained at -80°C until DNA extraction.

**Table 1:** Geographical coordinates of sampling sites.

Oil palm trees with Fatal Yellowing (Symptomatic Plants)		Oil palm trees with no signs of Fatal Yellowing (Asymptomatic plants)	
SP Stage 2 plant 1 –SP 2.1	S02°00’27.8” W048°38’18.3”	AP 1	S02°00’26.2” W048°35’52.3”
SP Stage 2 plant 2 –SP 2.2	S02°00’27.0” W048°38’18.1”	AP 2	S02°00’24.1” W048°35’52.4”
SP Stage 5 plant 1 –SP 5.1	S02°00’23.8” W048°38’18.6”	AP 3	S02°00’24.1” W048°35’52.3”
SP Stage 5 plant 2 –SP 5.2	S02°00’24.9” W048°38’17.6”	AP 4	S02°00’25.1” W048°35’51.4”
SP Stage 8 plant 1 –SP 8.1	S02°00’25.4” W048°38’16.9”		
SP Stage 8 plant 2 –SP 8.2	S02°00’26.8” W048°38’16.0”		

**SP**- FY Symptomatic Plants; **AP**- FY Asymptomatic Plants.

### Total DNA extraction

Total DNA was extracted according to Doyle [19], with modifications. All the leaflets from each plant were combined and ground in a mortar with liquid nitrogen. Two grams of plant material were then mixed with 3 g of acid washed glass beads (~ 200 μm), 5 mL of extraction buffer (2% CTAB, 1.4 M NaCl, 20 mM EDTA, 100 mM Tris-HCl pH8.0, 1% PVP) and 180 μL 20% SDS. Samples were incubated at 65 °C for 30 minutes, and then vortexed for 2 cycles of 90 seconds with 10 seconds intervals. After cooling, 4 mL of 25:24 –phenol/chloroform solution were added and samples were centrifuged at 2,880 x g for 8 minutes. The supernatant was then transferred to new tubes, 4 mL of chloroform-isoamyl alcohol (24: 1) were added and this mixture was centrifuged again as described above. The supernatant was transferred to new tubes and 2/3 volume of isopropanol was added. Samples were stored at -20°C for 16 hours and subsequently centrifuged for 10 minutes at 18,000 xg. After the supernatant was discarded, the pellet was washed with 2 mL of 70% ethanol and centrifuged again as before. The pellet was resuspended in 100 μL 10 mM Tris-HCl pH 8 in microfuge tubes. Next, 100 μg of RNAse were added and samples were incubated at 37 °C for 30 min. For DNA precipitation, 5 μL of 3 M sodium acetate pH 5.2, 50 μL 100 mM Tris-HCl pH 8.0 and 100 μL of cold 100% ethanol were added to the samples. Samples were kept at -20 °C for 10 min, and then centrifuged at 13,400 xg for 10 min. The supernatant was discarded, and the pellet was washed with cold 70% ethanol, centrifuged, dried and resuspended in 100–300 μL of 10 mM Tris-HCl pH 8.0.

### Amplification of *Phytophthora* genus ITS region

To investigate the presence of oomycete DNA of the genus *Phytophthora*, total extracted DNA was used for amplification by polymerase chain reaction (PCR) using primers specific for the genus *Phytophthora*, A2 (5’-ACT TTC CAC GTG AAC CGT TTC AA-3’) and I2 (5′ GAT ATC AGG TCC AAT TGA GAT GC 3′) [20]. Each 20 μL PCR reaction contained 1X reaction buffer (with 1.5 mM MgCl_2_), 0.25 mM each dNTP, 0.25 μM each primer, 1U Taq DNA Polymerase (Phoneutria, Minas Gerais, Brazil), 0.4 ng/μl bovine serum albumine and approximately 20 to 40 ng metagenomic DNA. Amplifications were performed in a GeneAmp^®^ PCR System 9700 thermal cycler (Applied Biosystems, California, USA). The following parameters were used: denaturation at 94° C for 5 minutes followed by 30 to 40 cycles of denaturation at 95 °C for 30 s, annealing at 60 °C for 30 seconds, and extension 72 °C for 1 min, with a final extension cycle at 72 °C for 4 minutes. As a positive control, genomic DNA of *Phytophthora nicotianae* (kindly provided by Dr. Café Filho, Department of Plant Pathology, University of Brasilia, Brazil) was used. Milli-Q water was used as a no DNA negative control.

### Amplification of fungal ITS region and bacterial 16S rRNA gene

Total DNA was used for amplification of the ITS region of the kingdom Fungi and the gene encoding the 16S ribosomal RNA of the *Bacteria* domain. The primers used were ITS1F (5′ CTT GGT CAT TTA GAG GAA GTA A 3′) [21] and ITS4 (5′ TCC TCC GCT TAT TGA TAT GC 3′) [22] for fungi; 799F (5′ AAC MGG ATT AGA TAC CCK G 3′) [23] and 1492R (5′ GGY TAC CTT GTT ACG ACT T 3′) [24] for *Bacteria*. Adapters used as priming sites for both amplification and sequencing (454 Life Sciences, Branford, CT, USA) were added to the 5′ end of the primer sequences (adapter A for forward primers, and adapter B for reverse primers). Amplifications with ITS1F/ITS4 primer pair were performed under the following conditions: denaturation at 95 °C for 30 s, followed by 40 cycles of denaturing at 95 °C for 30 s, annealing at 55 °C for 5 s and extension at 72 °C for 30 s, with a final extension cycle at 72 °C for 30 s. The conditions for PCR amplification with 799F/1492R primer pair were: denaturation at 95 °C for 30 s followed by 40 cycles of denaturing at 95 °C for 30 s, annealing at 53 °C for 5 s and extension at 72 °C for 30 s, with a final extension cycle at 72 °C for 30 s. Each 20 μL reaction contained approximately 20 ng of total DNA, 0.25 μM of each primer, 0.4 ng/μl bovine serum albumin and 10 μL Flash Phusion High-Fidelity PCR Master Mix (Thermo Fisher Scientific, Massachusetts, USA). As positive controls, genomic DNA of *Escherichia coli* and *Saccharomyces cerevisiae* were used for bacterial and fungal amplifications respectively. Milli-Q water was used as a no DNA negative control. Approximately 25 reactions were performed for each sample, and the PCR products were pooled and purified with the GeneJET PCR Purification Kit (Thermo Fisher Scientific, MA, USA). After purification, PCR products were quantified using the NanoDrop^™^ 1000 Spectrophotometer (Thermo Fisher Scientific, MA, USA). Pyrosequencing of the purified PCR products was performed on one-fourth of a sequencing plate using GS FLX Titanium platform at Macrogen, South Korea.

### Pyrosequencing analysis

Microbial community sequences were analyzed using the software package Mothur [25]. Denoising of the raw data was performed with the tool shhh.flows, an adaptation of the PyroNoise algorithm [26]. Multiplex identifier barcodes and adapters were removed, as well as sequences shorter than 250 bp. bacterial sequences were aligned using the align.seqs tool against the SILVA database (v128) [27]. Fungal sequences were aligned using the online alignment tool Multiple Alignment using Fast Fourier Transform (MAFFT) [28]. Chimeras were removed with the tool chimera.uchime. Phylogenetic classification of the microorganisms was performed using the SILVA database (v128) [27] for *Bacteria* and the UNITE database for Fungi [29]. The number of sequences in each sample was normalized to the smallest number of sequences in all groups. Sequences were clustered into operational taxonomic units (OTUs) using the average-neighbor method at a 3% distance threshold. Mothur was used to calculate diversity and richness indices and to produce Venn diagrams. Principal component analysis (PCA) was performed using the Stamp software [30], as well as analysis of variance (ANOVA), with the level of significance set at 0.05. For Venn diagrams, sample sequences were combined according to the stage of the disease or its absence, producing the groups SP 2 (samples SP 2.1 and 2.2), SP 5 (samples SP 5.1 and 5.2), SP 8 (samples SP 8.1 and 8.2) and AP (AP 7, 8, 9 and 10). Sequences belonging to OTUs exclusively shared by symptomatic samples (SP 2, SP5 and SP8) were initially classified with Mothur [25] using UNITE database [29] and then were analyzed using the Basic Local Alignment Search Tool (BLAST) [31], available online at the National Center for Biotechnology Information (NCBI) website.

## Results

Many tests were performed in an attempt to produce amplicons for the ITS region from the *Phytophthora* genus using as template total DNA extracted from diseased and apparently healthy plants. However, there was amplification in only one sample (SP 2.1) (S1 Fig). PCR using the same conditions were also performed with DNA from bulk soil collected in the same area, around diseased plants and native forest soil, provided by Tupinambá et al. [32], but no amplification occurred (S2 Fig).

Pyrosequencing of the fungal ITS region generated 232,869 raw sequences. After quality control and chimera removal, 136,615 sequences longer than 268 bp remained for subsequent analyses. Between 9,221 and 25,542 good quality reads were obtained per sample. We initially analyzed bacterial diversity, but after sequencing we observed a high percentage of chloroplast sequences in all the samples (34.7%-99% of the sequences), even though primer 799F [23], developed for endophytic studies, was used to avoid such sequences. Therefore, only the fungal diversity associated with the oil palm trees with and without FY was analyzed. Sequences were deposited in GenBank under the accession numbers MG244285—MG245771.

### Fungal diversity

**Fig 1:**
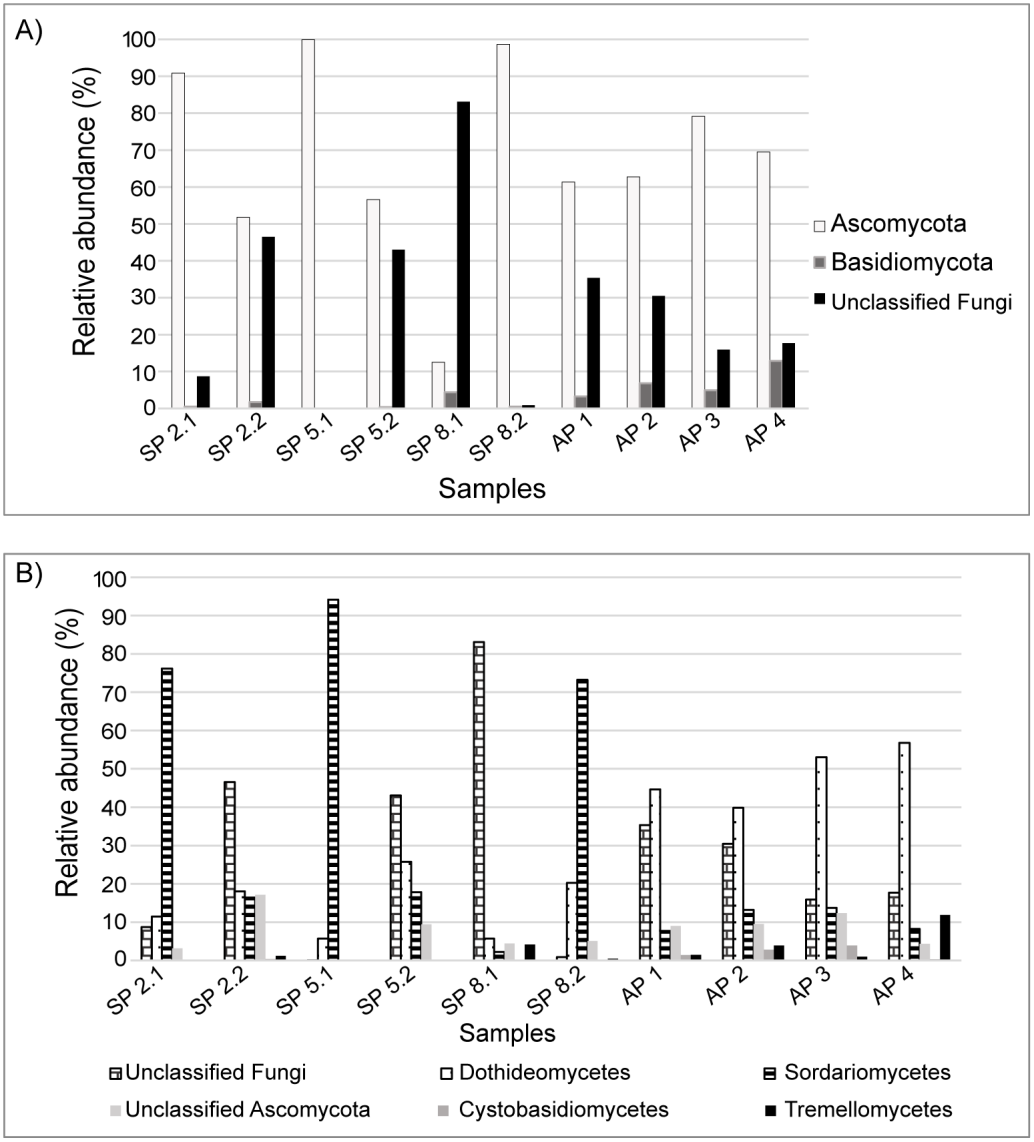
Sequence abundance at different taxonomic levels. Relative abundance of fungal sequences at phyla (A) and class (B) levels in oil palm leaves of plants with (stages 2, 5 and 8) or without Fatal Yellowing based on ITS region sequences. AP—FY Asymptomatic Plants; SP—FY Symptomatic Plants.

The number of sequences for the fungal ITS region after normalization was 9,221 sequences per sample (Table 2). Two phyla were observed in the samples (Ascomycota and Basidiomycota), as well as unclassified fungi (Fig 1A). The phylum Ascomycota was dominant in samples SP 2.1, SP 2.2, SP 5.1, SP 5.2, SP 8.2, AP 1, AP 2, AP 3 and AP 4 (51.8%–99.9% of the sequences), while unclassified fungi were predominant in SP 8.1 (83.1% of the sequences).

**Table 2:** Number of sequences, number of observed operational taxonomic units (OTUs), richness and diversity indices and coverage for the kingdom *fungi* in oil palm leaf samples at different stages of Fatal Yellowing (stages 2, 5 and 8) or in asymptomatic leaves (AP).

Sample	No. of seqs.*	OTUs	Chao1 index	ACE**	Shannon index	Inverse Simpson index	Good’s coverage estimator
**SP 2.1**	12,483	107	160.12	195.66	1.24	1.82	0.995
**SP 2.2**	17,025	166	203.27	203.02	2.83	6.24	0.996
**SP 5.1**	9,423	37	67.00	108.42	1.59	3.23	0.998
**SP 5.2**	11,374	155	236.48	307.45	2.29	4.46	0.994
**SP 8.1**	9,221	101	173.77	219.30	1.16	1.60	0.996
**SP 8.2**	9,759	88	217.38	342.83	2.14	6.05	0.995
**AP 1**	20,824	237	325.03	373.47	2.77	5.44	0.992
**AP 2**	10,874	213	328.56	278.66	3.09	7.74	0.994
**AP 3**	10,090	97	120.75	131.93	2.61	5.25	0.998
**AP 4**	25,542	126	180.08	208.85	2.30	3.66	0.995

* After chimaera removal.

**ACE: abundance-based coverage estimator.

At the class level, 16 groups were observed in total. Only unclassified fungi, Sordaryomicetes and Dothideomycetes were highly abundant in the samples (Fig 1B). Unclassified fungi were the most abundant in samples SP 2.2, SP 5.2 and SP 8.1 (43%–83.1% of the sequences). Sordariomycetes were predominant in samples SP 2.1, SP 5.1 and SP 8.2 (73.2%–94.2% of the sequences). Dothideomycetes were dominant in samples AP 1, AP 2, AP 3 and AP 4 (39.8%–56.8% of the sequences).

**Fig 2:**
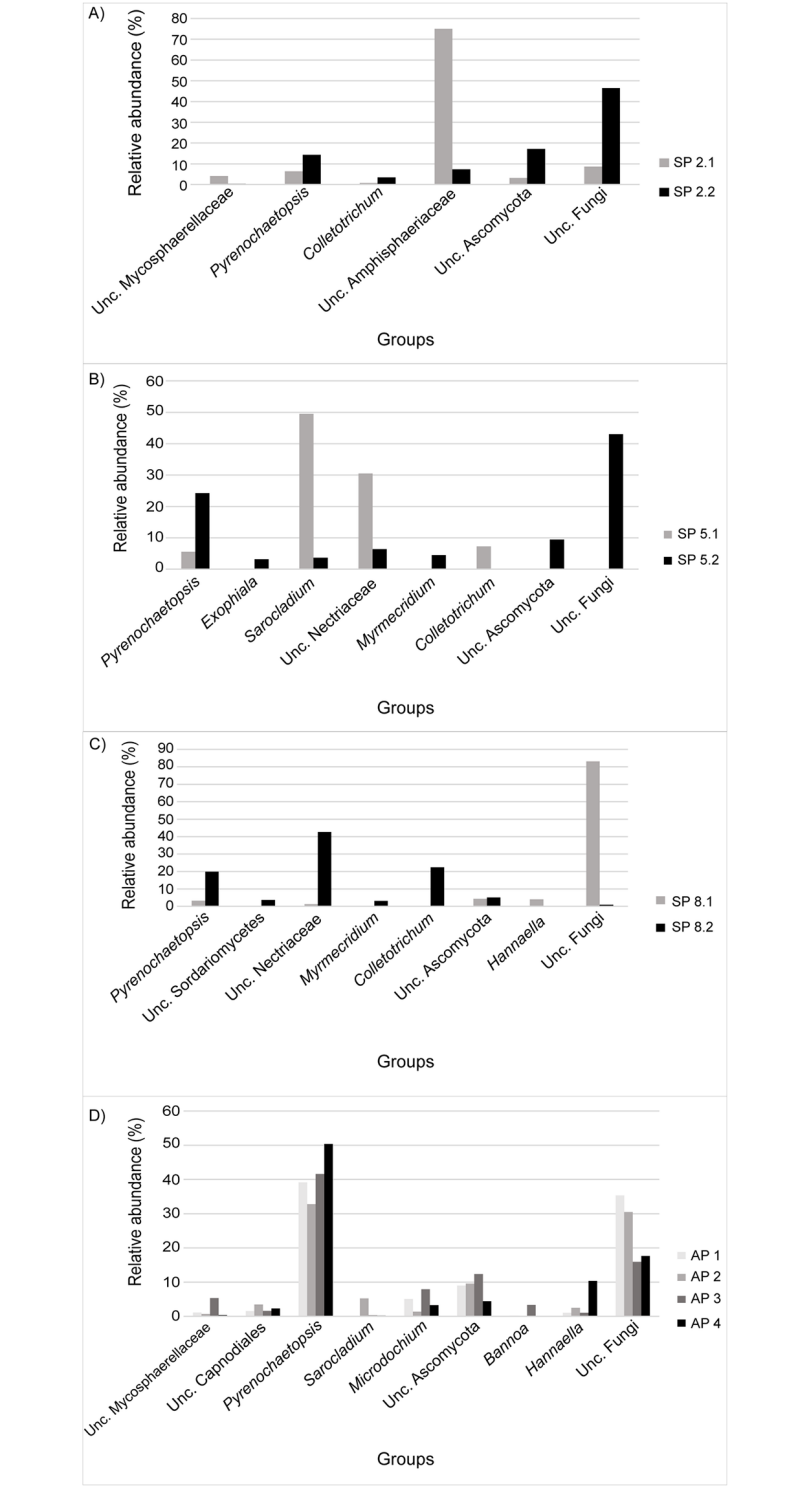
Fungal genera abundance. Relative abundance of fungal sequences at genus level in oil palm leaves of plants with (stages 2, 5 and 8) or without Fatal Yellowing based on ITS region sequences. (A) Stage 2 samples; (B) Stage 5 samples; (C) Stage 8 samples; (D) asymptomatic samples. AP—FY Asymptomatic Plants; SP—FY Symptomatic Plants.

At genus level, 97 groups were identified, including 34 unclassified groups and 61 known genera. As shown in Fig 2A–2D, ‘unclassified fungi’ was the most abundant group in samples SP 2.2, SP 5.2 and SP 8.1 (43%–83.1% of the sequences). ‘Unclassified Amphisphaeriaceae’ was the predominant group in sample SP 2.1 (75% of the sequences), while *Sarocladium* was the most abundant in SP 5.1 (49.5% of the sequences). *Pyrenochaetopsis* dominated AP 1, AP2, AP 3 and AP 4 (32.8%–50.4% of the sequences).

As shown on Table 2, the sample that had the largest amount of OTUs was AP 1 (237), and the one with the lowest number was SP 5.1 (37). Sample AP 1 also had the highest value for ACE richness estimator and sample AP 2 had the highest Chao1 estimator value. Sample SP 5.1 had the lowest Chao1 and ACE estimator values. For the diversity indices, sample AP 2 had the highest value for Shannon and inverse Simpson diversity estimators, and SP 8.1 had the lowest values for both estimators. Good’s coverage for all samples was above 99%, showing that the sampling effort was sufficient to cover the diversity of samples.

**Fig 3:**
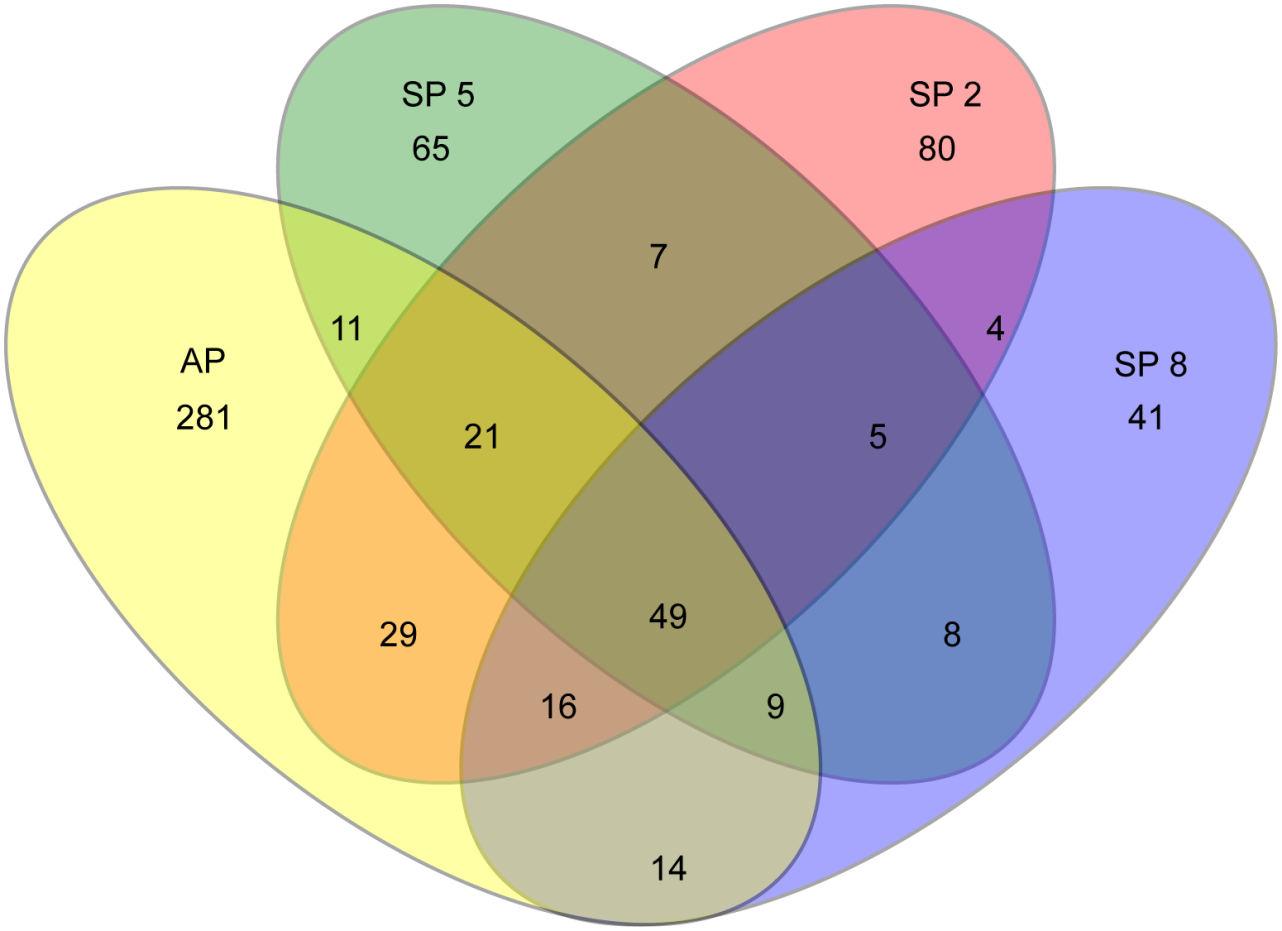
Venn diagram. The number of shared and unique fungal OTUs (at 3% distance) among groups of different stages of FY (stage 2, stage 5 and stage 8) and healthy plants (AP) is shown.

The Venn diagram for fungal OTUs at 0.03 distance (Fig 3) showed that AP group had the largest number of unique OTUs (281). Diseased plant groups (SP 2, 5 and 8) shared 5 OTUs, classified as ‘unclassified Ascomycota’ (OTU036 and OTU265), *Fusarium* sp. (OTU078), ‘unclassified Basidiomycota’ (OTU220) and ‘unclassified fungi’ (OUT164) (Table 3). After reclassification using BLAST, OTU036 and OTU220 have highest sequence identities with ‘unclassified uncultured fungus’, OTU078 is similar to *Fusarium solani*, OTU165 is similar to *Hannaella oryzae* and OTU265 is similar to *Colletotrichum citri*.

**Table 3:** Identification of fungal OTUs sequences shared by groups of oil palm leaf samples affected by FY in disease stages 2, 5 and 8, but not present in asymptomatic leaves.

Sequence ID	OTU number	Identity (%)	Genus and Genbank accession #
**JPNQ46G03CYILX**	OTU036	91%	Uncultured fungus KT328713.1
**JPNQ46G03CXECX**	OTU078	99%	*Fusarium solani* KF030977.1
**JPNQ46G03DPCVE**	OTU165	97%	*Hannaella oryzae* KU182505.1
**JPNQ46G03DAF46**	OTU220	94%	Uncultured fungus KU582345
**JPNQ46G03C3ZYL**	OTU265	100%	*Colletotrichum citri* KX670382.1

**Fig 4:**
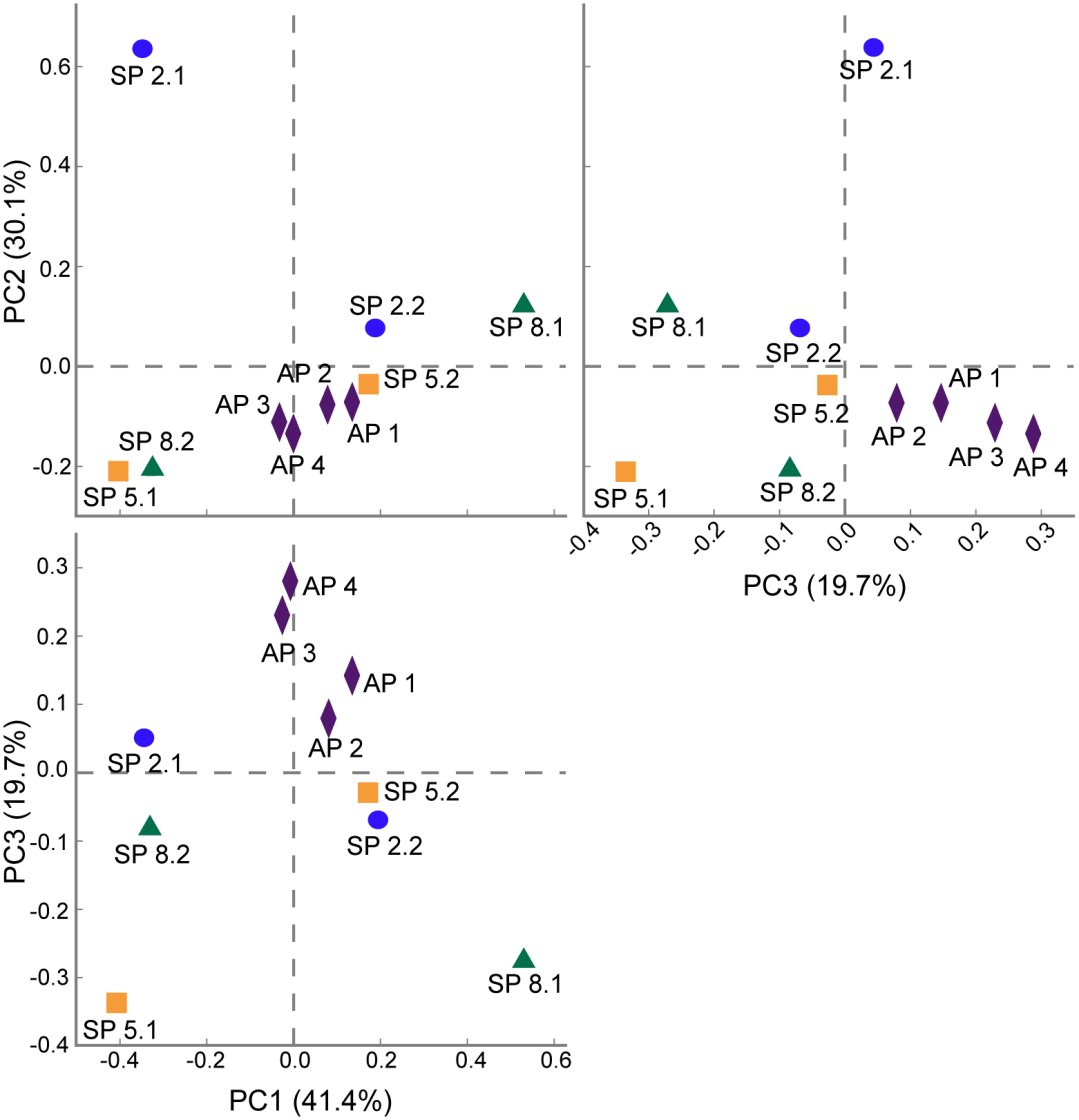
Genus-level principal component analysis (PCA) comparison of the fungal OTU data. Pairwise plots of the first three principal components based on multiple group analysis (PC1 and PC2 account for 71.5% of the variation across sites).

PCA (Fig 4) and ANOVA results (P-value > 0.05) showed that one fungal OTU caused the dispersion of the groups in the graph: *Pyrenochaetopsis* (higher relative abundance in AP group– 40.7%). PCA graphs also demonstrated that asymptomatic samples share more similarities, clustering together, while symptomatic samples are spread throughout the graph.

## Discussion

FY is an economically important disease that affects oil palm trees in Brazil. However, after many years of studies, its causal agent has not been determined. Abiotic and biotic factors have been correlated with the disease, and different possibilities have been explored as potential etiologic agents for FY. Insects [33], viroids and viroid-like RNA [34], nematodes [35] and phytoplasmas were evaluated, but none of these organisms had a direct relationship with the disease.

Fungi and other bacteria have also been related to FY. *Fusarium* sp., *Colletotrichum* sp., *Pestalotia* sp., *Rhizoctonia* sp., *Thielaviopsis* sp., *Nigrospora* sp., *Curvularia* sp., *Alternaria* spp., *Helminthosporium* sp., *Diplodia* sp., *Pestalotiopsis* sp., *Trichoderma* sp., *Pseudallescheria* sp. and *Lasiodiplodia* sp. were isolated from plants with FY, but none of them reproduced the disease upon inoculation [36]. Bacteria belonging to the genera *Erwinia*, *Pseudomonas* and *Bacillus* were also isolated from oil palm trees affected by *Pudrición del Cogollo*, but inoculation tests did not show development of the disease [17, 37].

In Colombia, however, Nieto [38] declared that PC was caused by a complex formed by *Thielaviopsis* sp., *Fusarium solani* and *Pythium*, concluding in another study that *Thielaviopsis* fulfilled the Koch Postulates and is the causal agent of the disease [38]. Also in Colombia, Sarría et al. [17] isolated *Phytophthora palmivora* from diseased oil palm trees and after pathogenicity tests, stated that this oomycete was responsible for the initial lesions of PC. After that, Martinez et al. [16], re-isolated *P*. *palmivora* from oil palm trees and reaffirmed that *P*. *palmivora* is the causal agent of PC. Torres et al. [39] and Sarría-Villa et al. [40] reported then that *P*. *palmivora* fulfilled Koch’s postulates and affirmed once more that it is the causal agent of *Pudrición del Cogollo* in Colombia. Moreover, Torres et al. [41] believe that the initial development of bud rot lesions in oil palm trees can be related to insects from family Tettigoniidae, through the production of lesions in the spear leaves and transport of reproductive structures of *P*. *palmivora*. In Ecuador, however, Narváez [42] looking for microorganisms in oil palm trees with PC, did not isolate *P*. *palmivora* and did not detect it by DAS-ELISA. It was detected only in two samples, with a serological strip.

In this work, we initially investigated whether *P*. *palmivora* could be the causal agent of FY in oil palm plants in Brazil. To this end, genus-specific primers for *Phytophthora* were used in an attempt to amplify the DNA of the oomycete from leaves of oil palm trees in three stages of the disease, and in plants with no apparent symptoms. Amplification happened only in one sample, similar to the study of Narváez [42] where immunological strips detected *Phytophthora* genus’ proteins in just 2 of the 33 trees sampled. In our work, it is not possible to state that the DNA amplified is derived specifically from *P*. *palmivora*, since the primer pair used was not specific for the species. Amplification did not happen either when DNA from soil collected around oil palm trees with and without symptoms of the disease was used. In addition, *P*. *palmivora* was never isolated from oil palm trees with FY in Brazil [43]. These data support the findings of Ayala [13], Zadoks [15] and Swinburne [14], who state that FY in Brazil and PC in Colombia are different diseases. In Brazil, *P*. *palmivora* has been related to root and fruit rot of papaya [44], but not FY.

Even though FY has been studied for many years, no work used high throughput sequencing to describe the microbial community associated with leaves of diseased plants, or even healthy oil palm trees. Most of the works related to palm trees focus on fruits, seeds, roots and bulk soil since these are economically important to agroindustry [45]. Tupinambá et al. [32], however, used pyrosequencing to study archaeal communities in Amazon forest soil cultivated with oil palm trees. In addition, most of the studies that evaluate microorganisms associated with palm trees describe the isolation of fungi recovered from unhealthy plants by classical microbiology techniques, in an attempt to find the causal agent of palm tree diseases.

Rodrigues [46] studied fungal endophytes from leaves and saplings of the Amazonian palm *Euterpe oleraceae* and isolated 57 species. The most common were *Xylaria cubensis* and *Letendraeopsis palmarum*. The author concluded that different regions of the palm tree had different endophytic communities. Fróhlich et al. [47] isolated 48 genera of endophytic fungi associated to six *Licuala* sp. palms and observed that the communities were dominated by *Xylaria* sp. Treu [48] isolated approximately 50 species of fungi collected in oil palm plantations in South East Asia. Among the genera identified there were *Ganoderma*, *Humphreya*, *Picnoporus*, *Rigidoporus*, *Gymnopilus* and *Pleutorus*. Rungjindamai et al. [49] isolated, in Thailand, 340 endophytes from healthy leaves, rachis and petioles of *Elaeis guineensis*. The authors then characterized 13 basidiomycetes, which belonged to the genus *Schizophyllum*, *Pycnoporus* and *Fomitopsis*. Later, Pinruan et al. [45] characterized seven more of these isolates, which were identified as *Schizophyllum* (1), *Pycnoporus* (2), *Fomitopsis* (1), *Trametes* (2), and *Perenniporia* (1).

Pyrosequencing generated 136,615 good quality sequences for the ITS region. The number of ITS sequences was enough to cover more than 99% of the diversity for all samples. Even though asymptomatic plants were used for comparison in this work, it is not possible to know that these trees were completely free of the disease, since the causal pathogen is unknown. However, samples of asymptomatic leaves were collected 4 km away from diseased plants, in an area apparently free of FY.

In this study, at genus level, different groups predominated in all FY symptomatic samples (SP2.1-SP 8.2) (Fig 2A, 2B and 2C), while two groups (*Pyrenochaetopsis* and unclassified fungi) predominated in all asymptomatic samples (AP1-AP 4) (Fig 2D). The fungal communities were not consistent between samples at the same stage of the disease or among all the symptomatic samples. This tendency was confirmed by PCA analysis, where asymptomatic samples (AP 1- AP4) were close to each other, while symptomatic samples (SP2.1-SP 8.2) did not form clusters (Fig 4). The fungal community in symptomatic plants is not stable, and no fungal taxon had its relative proportion increased across all the diseased plants. This result suggests that the as the disease progresses, different microorganisms may predominate, thus the microbial communities present are not the same in plants at the same disease stage. Therefore, the changes observed in the fungal composition may be a secondary effect of the disease, not the primary cause.

At stage 2 (Fig 2A), unclassified *Amphisphaeriaceae* (SP 2.1) and unclassified fungi (SP 2.2) were predominant. *Amphisphaeriaceae* is an important ascomycete family belonging to the *Xylariales* order that comprises weak plant pathogens and some endophyte species [50]. Quadros et al. [51] evaluated the genetic diversity of fungi from the family *Amphisphaeriaceae* in oil palm trees in Pará state, Brazil. The authors isolated nine fungi from oil palm tree leaves with FY that displayed characteristic lesions of the genus *Pestalotiopis*, and sequenced the ITS region of the isolates. After sequence analysis, it was observed that the isolates belonged to the genus *Pestalotiopsis*, *Neopestalotiopsis* and *Pseudopestalotiopsis*. The presence of lesions caused by these genera of fungi seems to be common in oil palm plantations, as well as the presence of *Amphispaeriaceae* family in general [51]. Boari [36], however, observed that *Pestalotiopsis* was not capable of causing FY when inoculated in palm oil trees.

At stage 5 (Fig 2B), *Sarocladium* (SP 5.1) and unclassified fungi (SP 2.2) were the most abundant. *Sarocladium* is a fungal genus that encompasses approximately 10 species, including traditionally important plant pathogens and opportunistic human pathogens. The type species of the genus is *Sarocladium oryzae*, that causes the sheath rot of rice [52].

At stage 8 (Fig 2C), unclassified fungi (SP 8.1) and unclassified *Nectriaceae* (SP 8.2) were the predominant groups. The family Nectriaceae includes several important plant and human pathogens, as well as species explored in the industry as biodegraders and biocontrol agents. Most of these fungi are soil-borne saprobes, weak to virulent, facultative to obligatory plant pathogens [53].

In addition to the most abundant groups, *Colletotrichum*, an important plant pathogen, appears as predominant group in diseased samples SP 2.2, 5.1 and 8.2 (Fig 2), and it is closely related to one of the OTUs that are only present in diseased samples (Table 3). This genus contains pathogens that cause anthracnose in several woody and herbaceous plants [54]. Another plant pathogen found among the OTUs only present in diseased samples is *Fusarium*, a genus that causes economically important blights, root rots and wilts in a wide range of plant species [55]. Common fungi found in leaf surfaces, such as *Hannaella* and *Exophiala* [56], were also observed (Fig 2). The amount of unclassified sequences in all the samples shows that there is still a huge unknown fungal diversity in oil palm trees, despite all the culture-based studies. Among the predominant genus in symptomatic samples, *Colletotrichum*, *Fusarium* and *Pestalotiopsis* (*Amphisphaeriaceae)*, even though pathogenic, have already been inoculated in oil palm trees, but did not reproduce the disease [43]. Even though they were ruled out as the etiologic agents of FY, these genera could be a secondary effect of the disease, opportunistic pathogens increasing the severity of the infection. The genus *Sarocladium* includes important plant pathogens, however, its isolation from oil palm trees with FY has not yet been reported. In addition, there are numerous unclassified sequences in all the samples, symptomatic or asymptomatic. Therefore, the causal agent of FY could be still among genera that are yet unknown.

In the asymptomatic samples, *Pyrenochaetopsis* and unclassified fungi were the most abundant groups (Fig 2d). *Pyrenochaetopsis* was introduced to accommodate several species formerly classified as *Phoma* and *Pyrenochaeta* which are soil borne and mainly associated to gramineous plants [57]. This genus has been found as endophytic in healthy leaves of *Cocos nucifera* [58].

Venn graphs for fungal OTUs (Fig 3) showed that the number of exclusive fungal OTUs to a specific disease stage, compared to asymptomatic group of samples (AP-281 exclusive OTUs), decreased with the progression of the disease (i.e., from 80 exclusive OTUs in stage 2 to 41 OTUs in stage 8). This type of dynamics is similar to the succession of communities found in decaying wood systems, where in later stages of decomposition, the fungal activity declines and the diversity of the community decreases [59, 60]. Among the OTUs that are not present in asymptomatic samples and are shared by symptomatic samples of all disease stages, only three could be classified, belonging to the genus *Fusarium*, *Colletotrichum* and *Hannaella* (Table 3). Both *Fusarium* and *Colletotrichum* have been inoculated in oil palm trees, and the plants did not develop FY [37, 43]. The genus *Hannaella* has not yet been related to plant diseases, being commonly found in leaf surfaces [61]. Two of the FY exclusive OTUs were not classified as any known genera; however, to be assigned as the causal agent of FY, Koch’s postulate must be fulfilled, where these microorganisms should be isolated, characterized and after inoculation into healthy oil palm trees the disease should be reproduced.

These results suggest that with the progress of the disease, decayed plant material produced by the primary colonizer species can generate metabolites that select the successive species, causing changes in the fungal community. The later composition of the fungal communities depends on the sequence of species that establishes a habitat in the plant material, since the production of metabolites can facilitate or prevent the development of later-arriving species [62, 63]. Colonization by different species in the first lesions of the disease in each plant could explain the later variation of the fungal communities between plants in the same disease stage. However, the pathogen that produces the primary lesions has still to be determined.

Since the variation of the microbial community depends on which microorganism establishes habitat first, the best approach to unravel the causal agent of the FY would be to combine classical microbiology and molecular biology to study the earlier stages of the disease. In late stages of the disease plant tissue is most likely invaded by opportunistic microorganisms.

## Conclusions

Oil palm is an economically important crop with potential for biodiesel production that is affected by a disease known as Fatal Yellowing. After many years of study, the causal agent of FY has not been unequivocally determined. Even though the cause of the *Pudrición del Cogollo* in Colombia was attributed to *Phytophthora palmivora*, it was never isolated from oil palm trees with FY in Brazil. In this study, the DNA of the genus *Phytophtora* was amplified from only one sample out of 10, further supporting the idea that FY in Brazil and *Pudrición del Cogollo* in Colombia are not the same disease, and that FY is not caused by *P*. *palmivora*. This is the first work to use high throughput next generation sequencing to evaluate the fungal community associated with leaves of oil palm trees with and without symptoms of Fatal Yellowing. Analyses of ITS region sequences demonstrated that symptomatic trees do not have a common microbial community, showing that the disease most likely causes a disturbance of the microbial community, with the presence of several opportunistic pathogens in the later stages. Even though it is not possible to confirm that the asymptomatic plants are completely free of the disease, the communities in the asymptomatic samples are more similar to each other in composition than the symptomatic samples. This is an interesting finding given that this is the first study that describes how a fungal community evolves in the course of plant disease. Further studies that explore other taxonomic groups (i.e., *Bacteria* and *Archaea*) as the possible etiologic agent for FY should be performed using plants in the initial stages of the disease, since in later stages the plants seem to be dominated by opportunistic pathogens.

## Supporting information

S1 Fig 1% agarose gel electrophoresis stained with ethidium bromide. Amplification with primer pair A2/I2. **1**^**st**^ **row. L**- Ladder 1 kb plus (Invitrogen). **1**-SP 2.1; **2**-SP 2.1 diluted 10X; **3**-SP 2.2; **4**-SP 2.2 diluted 10X; **5**-SP 5.1; **6**-SP 5.1 diluted 10X; **7**-SP 5.2; **8**-SP 5.2 diluted 10X; **9**-SP 8.1; **10**-SP 8.1 diluted 10X; **11**-SP 8.2.; **12**-SP 8.2 diluted 10X; **13**- AP 1; **14**-AP 1 diluted 10X. **2**^**nd**^ **row**. **L**- Ladder 1 kb plus (Invitrogen). **1**-AP 2; **2**-AP 2 diluted 10X; **3**-AP 3; **4**-AP 3 diluted 10X; **5**-AP 4; **6**-AP 4 diluted 10X; **7**-*Phytophthora* sp. genomic DNA; **8**-*Phytophthora capsicii* genomic DNA; **9**-*Phytophthora nicotianae* genomic DNA; **10**-*Phytophthora* sp. genomic DNA; **11**-*Escherichia coli* genomic DNA; **12**-Positive control (*Phytophthora nicotianae* genomic DNA); **13**-Negative control (water); **14**-empty.
(TIF)

S2 Fig 1% agarose gel electrophoresis stained with ethidium bromide. Amplification with primer pair A2/I2. **L**- Ladder 1 kb plus (Invitrogen). **1**-Soil asymptomatic plant diluted 5X; **2**- Soil asymptomatic plant diluted 15X; **3**-Soil asymptomatic plant diluted 100X; **4**-Soil symptomatic plant stage 5 diluted 5X; **5**-Soil symptomatic plant stage 5 diluted 15X; **6**-Soil symptomatic plant stage 5 diluted 100X; **7**-Soil symptomatic plant stage 8 diluted 5X; **8**- Soil symptomatic plant stage 8 diluted 15X; **9**-Soil symptomatic plant stage 8 diluted 100X; **10**-Native Forest soil; **11**-(*Phytophthora nicotianae* genomic DNA); **12**-Negative control (water); **13**-empty; **14**-empty.
(TIF)
