# Correction: Fungal diversity in oil palm leaves showing symptoms of Fatal Yellowing disease

**DOI:** 10.1371/journal.pone.0254042

**Published:** 2021-06-28

**Authors:** Ohana Yonara de Assis Costa, Daiva Domenech Tupinambá, Jessica Carvalho Bergmann, Cristine Chaves Barreto, Betania Ferraz Quirino

After this article [1] was published, concerns were raised about the lack of internal amplification controls in the *Phytophthora* PCR experiments reported in S1 Fig and S2 Fig. The experiments included independent positive and negative controls (see lanes 7–12 of the lower panel in S1 Fig, and lanes 11–12 of S2 Fig), but did not include positive amplification controls for each of the DNA samples used as experimental inputs.

The corresponding author stands by the published results. She agrees that formal amplification controls are lacking, but noted that the same samples were used in PCR-amplifications to generate all the data reported on the paper. The samples needed to repeat the PCR experiments with additional controls are no longer available.

A member of *PLOS ONE*’s Editorial Board advised that the concerns raised are valid, and that the PCR experiments did not have sufficient controls to support claims about the lack of *Phytophthora* in the samples. This impacts the interpretation of results shown in S1 Fig and S2 Fig, and the reliability of statements in paragraph 1 in the Results section, paragraph 4 of the Discussion, and in the Conclusions.

In light of this issue, sentence 4 of the article’s Conclusions is updated as follows:

Original version*:

“In this study, the DNA of the genus *Phytophtora* was amplified from only one sample out of 10, further supporting the idea that FY in Brazil and Pudrición del Cogollo in Colombia are not the same disease, and that FY is not caused by *P*. *palmivora*.”

Updated version:

“This study provided preliminary evidence that DNA of the genus *Phytophtora* may not be commonly present in Brazilian FY, contrary to what has been reported in Colombia. Further experiments with additional controls are needed to clarify the validity of this observation.”

*Note, the original Conclusion statement cited *Phytophthora* amplification in one out of 10 samples, but only 6 of the 10 samples assayed were from symptomatic plants.

The above issues do not impact the article’s results and conclusions that focus on fungal diversity in oil palm leaves with symptoms of Fatal Yellowing disease (FY).
