# Supplementary figures and images for: Fungal diversity in oil palm leaves showing symptoms of Fatal Yellowing disease
Source: PLoS One. 2018 Jan 25;13(1):e0191884. doi: 10.1371/journal.pone.0191884 (PMC5785003; doi:10.1371/journal.pone.0191884)

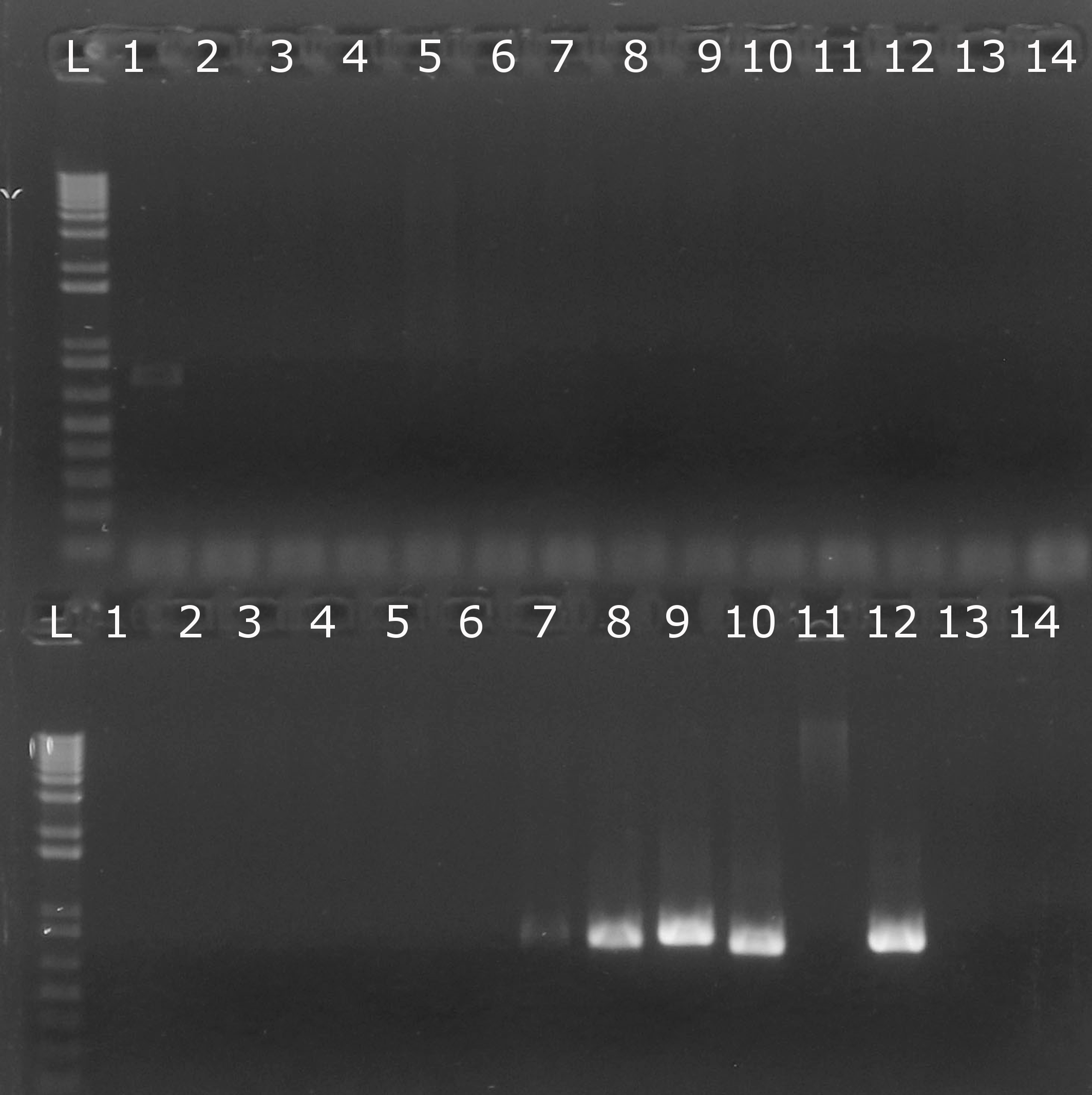

Supplement: S1 Fig — Amplification with primer pair A2/I2. 1st row. L- Ladder 1 kb plus (Invitrogen). 1-SP 2.1; 2-SP 2.1 diluted 10X; 3-SP 2.2; 4-SP 2.2 diluted 10X; 5-SP 5.1; 6-SP 5.1 diluted 10X; 7-SP 5.2; 8-SP 5.2 diluted 10X; 9-SP 8.1; 10-SP 8.1 diluted 10X; 11-SP 8.2.; 12-SP 8.2 diluted 10X; 13- AP 1; 14-AP 1 diluted 10X. 2nd row. L- Ladder 1 kb plus (Invitrogen). 1-AP 2; 2-AP 2 diluted 10X; 3-AP 3; 4-AP 3 diluted 10X; 5-AP 4; 6-AP 4 diluted 10X; 7-Phytophthora sp. genomic DNA; 8-Phytophthora capsicii genomic DNA; 9-Phytophthora nicotianae genomic DNA; 10-Phytophthora sp. genomic DNA; 11-Escherichia coli genomic DNA; 12-Positive control (Phytophthora nicotianae genomic DNA); 13-Negative control (water); 14-empty. (TIF) [file pone.0191884.s001.tif]

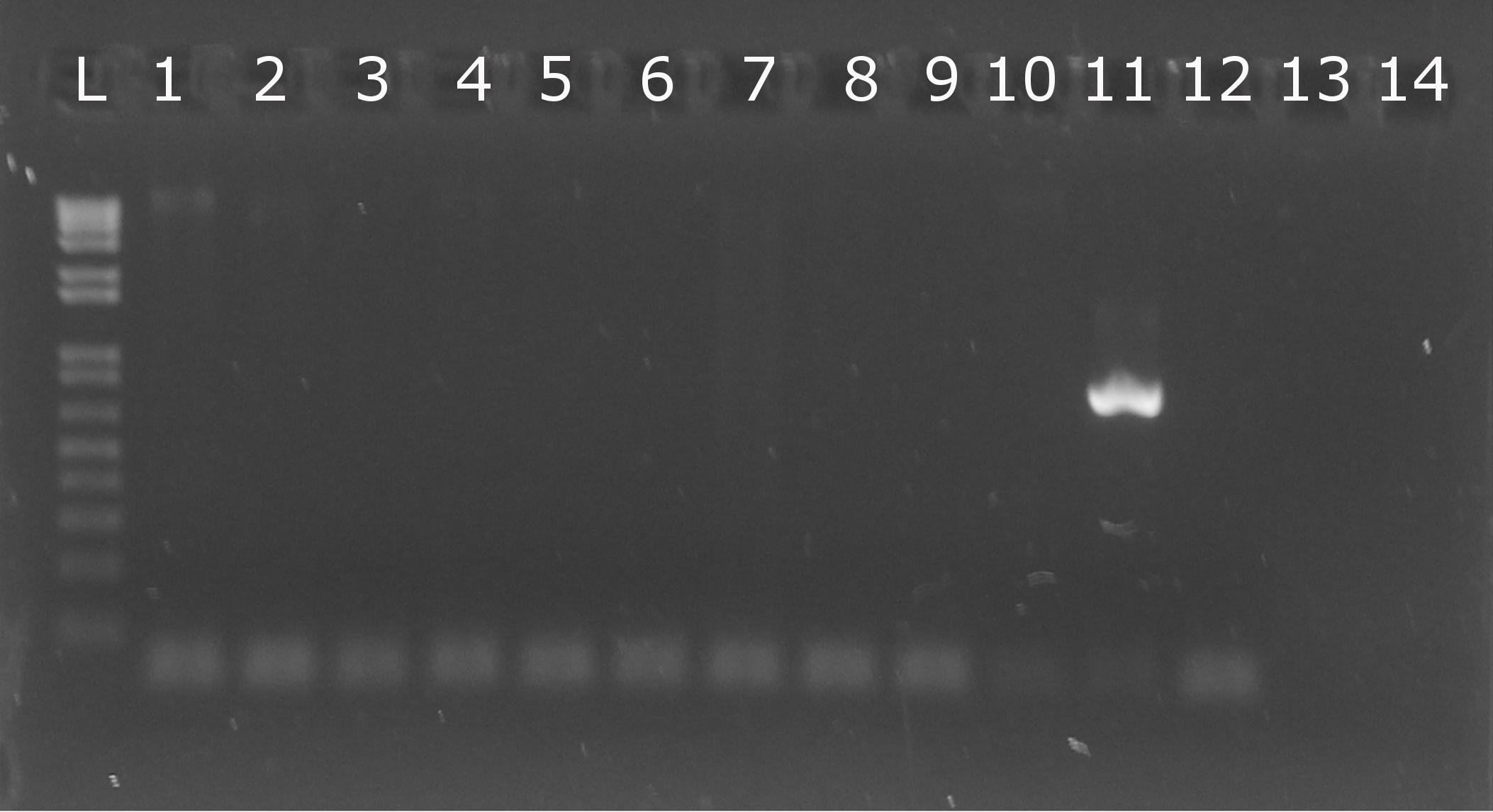

Supplement: S2 Fig — Amplification with primer pair A2/I2. L- Ladder 1 kb plus (Invitrogen). 1-Soil asymptomatic plant diluted 5X; 2- Soil asymptomatic plant diluted 15X; 3-Soil asymptomatic plant diluted 100X; 4-Soil symptomatic plant stage 5 diluted 5X; 5-Soil symptomatic plant stage 5 diluted 15X; 6-Soil symptomatic plant stage 5 diluted 100X; 7-Soil symptomatic plant stage 8 diluted 5X; 8- Soil symptomatic plant stage 8 diluted 15X; 9-Soil symptomatic plant stage 8 diluted 100X; 10-Native Forest soil; 11-(Phytophthora nicotianae genomic DNA); 12-Negative control (water); 13-empty; 14-empty. (TIF) [file pone.0191884.s002.tif]
